# Factors Predicting and Reducing Mortality in Patients with Invasive *Staphylococcus aureus* Disease in a Developing Country

**DOI:** 10.1371/journal.pone.0006512

**Published:** 2009-08-04

**Authors:** Emma K. Nickerson, Vanaporn Wuthiekanun, Gumphol Wongsuvan, Direk Limmathurosakul, Pramot Srisamang, Weera Mahavanakul, Janjira Thaipadungpanit, Krupal R. Shah, Arkhom Arayawichanont, Premjit Amornchai, Aunchalee Thanwisai, Nicholas P. Day, Sharon J. Peacock

**Affiliations:** 1 Mahidol-Oxford Tropical Medicine Research Unit, Mahidol University, Bangkok, Thailand; 2 Centre for Clinical Vaccinology and Tropical Medicine, Nuffield Department of Clinical Medicine, University of Oxford, Oxford, United Kingdom; 3 Department of Paediatrics, Sappasithiprasong Hospital, Ubon Ratchathani, Thailand; 4 Department of Medicine, Sappasithiprasong Hospital, Ubon Ratchathani, Thailand; 5 Duke University Medical Center, Durham, North Carolina, United States of America; 6 Department of Medicine, University of Cambridge, Addenbrooke's Hospital, Cambridge, United Kingdom; Columbia University, United States of America

## Abstract

**Background:**

Invasive *Staphylococcus aureus* infection is increasingly recognised as an important cause of serious sepsis across the developing world, with mortality rates higher than those in the developed world. The factors determining mortality in developing countries have not been identified.

**Methods:**

A prospective, observational study of invasive *S. aureus* disease was conducted at a provincial hospital in northeast Thailand over a 1-year period. All-cause and *S. aureus*-attributable mortality rates were determined, and the relationship was assessed between death and patient characteristics, clinical presentations, antibiotic therapy and resistance, drainage of pus and carriage of genes encoding Panton-Valentine Leukocidin (PVL).

**Principal Findings:**

A total of 270 patients with invasive *S. aureus* infection were recruited. The range of clinical manifestations was broad and comparable to that described in developed countries. All-cause and *S. aureus*-attributable mortality rates were 26% and 20%, respectively. Early antibiotic therapy and drainage of pus were associated with a survival advantage (both p<0.001) on univariate analysis. Patients infected by a PVL gene-positive isolate (122/248 tested, 49%) had a strong survival advantage compared with patients infected by a PVL gene-negative isolate (all-cause mortality 11% versus 39% respectively, p<0.001). Multiple logistic regression analysis using all variables significant on univariate analysis revealed that age, underlying cardiac disease and respiratory infection were risk factors for all-cause and *S. aureus-*attributable mortality, while one or more abscesses as the presenting clinical feature and procedures for infectious source control were associated with survival.

**Conclusions:**

Drainage of pus and timely antibiotic therapy are key to the successful management of *S. aureus* infection in the developing world. Defining the presence of genes encoding PVL provides no practical bedside information and draws attention away from identifying verified clinical risk factors and those interventions that save lives.

## Introduction

*Staphylococcus aureus* is increasingly recognised as an important cause of serious sepsis in the developing world, where the associated mortality far exceeds that in developed countries [1]–[3]. Adverse outcomes are also observed in countries with growing economies where available healthcare facilities may vary considerably between major cities and provincial or rural areas. For example, death attributable to *S. aureus* bacteraemia in a large, 1,000 bed provincial hospital in Thailand was recently reported to be 48% [1], roughly double that for well resourced healthcare settings in Europe and the United States [4]–[7]. Factors associated with poor outcome from *S. aureus* infection in the developed world include increasing age [6], [8], underlying co-morbidities [9], antimicrobial resistance [10], complicated (disseminated) bacteraemia [11], lack of source control [8] including non-removal of intravenous catheters [12], under-dosing of penicillinase antibiotics for methicillin-susceptible *S. aureus* (MSSA) [8], and delayed antibiotic therapy [13]. The relevance of these findings for the developing world is not directly addressed in the published literature, but it is likely that low-cost interventions such as timely administration of antibiotics and drainage of pus which are advocated as the standard of care elsewhere [14], [15] would reduce poor outcomes in developing country settings. The first aim of this observational study was to identify risk factors for death from *S. aureus* infection and determine the effect on outcome of simple clinical interventions in a provincial hospital in a lower-middle income setting in Asia.

Panton-Valentine Leukocidin (PVL) is a bicomponent cytotoxin and a putative *S. aureus* virulence factor that has been associated with skin and soft tissue infections [16], [17] together with more severe manifestations including necrotising pneumonia [18], [19]. PVL has been strongly associated with the emergence of community-acquired methicillin-resistant *S. aureus* (CA-MRSA) [20], [21]. Its role in virulence is currently the subject of much research and debate within the staphylococcal community, with some researchers calling for rapid tests to detect the presence of genes encoding PVL [22], [23]. The second aim of this study was to undertake a cohort study to determine the role of PVL as a risk factor for death in unselected patients with *S. aureus* infection.

## Methods

### Participants and clinical methods

Ethical approval was obtained from the Ethical and Scientific Review sub-committee of the Royal Thai Government Ministry of Public Health and the Oxford Tropical Research Ethics Committee. A prospective, observational study was conducted at Sappasithiprasong Hospital in northeast Thailand for a period of 1 year, from November 2006 to November 2007. This 1,000-bed regional hospital serves a catchment of 2 million people and provides a comprehensive clinical and laboratory service. Potential study patients were identified by daily consultation with the hospital diagnostic microbiology laboratory. Patients of any age with at least one sample taken from a normally sterile site positive for a pure growth of *S. aureus* were considered for inclusion. Blood culture was considered to represent a sterile site sample, and other sample types were assessed based on details in the clinical notes and discussion with nursing staff. Surface swabs and other samples not collected from usually sterile sites or not obtained via aspiration or an operative procedure were excluded. Patients were enrolled into the study after written informed consent was obtained, and were visited daily by study investigators until discharge to record progress and management. Data were recorded on standardised forms adapted from those used by Fowler et al [7]. Final outcome was determined 12 weeks from the date the first culture positive for *S. aureus* was taken using a standardised telephone questionnaire. Some data for the subset of patients with bacteraemia has been published elsewhere [3].

### Definitions

Community-acquired infection was defined as a positive *S. aureus* sterile site culture and admission to hospital with an illness consistent with invasive *S. aureus* disease. Nosocomial infection was defined as a positive *S. aureus* sterile site culture taken more than 48 hours after admission for another condition. Non-nosocomial healthcare-associated infection was defined as community-acquired infection in an individual who had contact with healthcare services in the preceding year, using the criteria described by Fowler et al [7]. Outcomes at 12 weeks were defined as: (i) cure - clinically improved and no additional sites of infection present or suspected; (ii) unresolved infection - persistent features of infection with or without persistent positive cultures; (iii) death attributable to *S. aureus* - when death was due to *S. aureus* infection in a previously healthy individual or when *S. aureus* hastened death in the presence of an underlying condition such as cancer; or (iv) death due to other causes - in which the *S. aureus* infection did not appear to contribute to death.

### Laboratory Methods

The hospital diagnostic microbiology laboratory processed all samples using routine procedures, including antibiotic susceptibility testing using the disk diffusion method [24]. Susceptibilities reported to hospital physicians were penicillin, oxacillin, cefazolin, erythromycin, clindamycin and trimethoprim-sulphamethoxazole for methicillin-susceptible *S. aureus* (MSSA), plus vancomycin and fusidic acid for methicillin-resistant *S. aureus* (MRSA). A total of 248 *S. aureus* isolates (first positive sample for each patient, with preference given to blood culture samples when two different samples taken on the same day were both positive) were available and obtained by the investigators, re-identified and stored at −80°C. An extended antibiotic susceptibility profile to cefoxitin, chloramphenicol, ciprofloxacin, clindamycin, erythromycin, fusidic acid, gentamicin, mupirocin, netilmicin, penicillin, rifampicin, trimethoprim-sulphamethoxazole, tetracycline and vancomycin was defined using the disk diffusion method [24]. Isolates that were resistant to cefoxitin by disk diffusion were further evaluated by oxacillin and vancomycin E-tests (AB Biodisk). Isolates were designated as MRSA based on an oxacillin E-test for 248 isolates re-tested in the research laboratory, and based on the oxacillin disk diffusion assay for 22 isolates that were tested by the hospital laboratory but were not available for further testing.

A multiplex polymerase chain reaction (PCR) was used to determine the presence of *mecA*, the genes encoding PVL and the 16S rRNA gene (internal positive control). A 1 ml overnight culture of *S. aureus* with an optical density of 1.0 at 600 nm was centrifuged at 16,000×g for 30 seconds and the cell pellet re-suspended with 200 µl phosphate buffer solution (pH 7). Genomic DNA was extracted using the High Pure PCR Template Preparation Kit (Roche Applied Science, Germany), with an additional step of incubation at 37°C for 30 minutes with 0.5 µl of 10 mg/ml lysostaphin solution (Sigma, USA) prior to cell lysis. The primers used were as described previously for *mecA* (primers mA1 and mA2) [25], the PVL genes (*lukS-PV* and *lukF-*PV) [18], and the 16S rRNA gene (Staph756F and Staph750R) [26] which is genus-specific and was used as a positive control for each reaction. A 25 µl PCR reaction was used containing 10–120 ng genomic DNA, 5pmol of each primer (Proligo Singapore Pty Ltd), 200 µM dNTP (Qiagen, Germany), 1.25 units of Taq polymerase (Roche Applied Science, Germany), 4.5 mM MgCl_2_ and 1×buffer. PCR was performed using a PTC-200 Peltier Thermal Cycler (MJ research, USA). The PCR conditions were one cycle of 95°C for two minutes, 30 cycles of 15 seconds at 95°C, 15 seconds at 56°C, 45 seconds at 72°C, and a final step of 72°C for seven minutes. Amplification product size was determined by reference to a 100-bp molecular weight marker (Biolabs New England, UK) using 2% agarose gel electrophoresis.

### Statistical analysis

Data was double entered into a database. Data analyses were performed using Stata software version 9 (StataCorp, College Station, Texas). Categorical data were compared using Fisher's exact test and for continuous data Student's t test was used. Variables significant at p<0.20 on univariable analysis and with 5 or more events (deaths) were used in the multivariable logistic regression model. Superficial and deep abscesses were combined so that there were 5 events. Prematurity was excluded given the low number of cases overall (n = 7). Variables which did not reach this threshold were reviewed and those considered a priori to potentially have an important influence on outcome were added (diabetes mellitus was the only variable in this category). Stepwise removal and addition of variables were used to determine the final multivariable model (p for removal 0.1 and p for re-entry 0.05). Missing PVL data was excluded from the multivariable logistic regression. Potential interactions were assessed using logistic regression. Reported p values are two-tailed.

## Results

During the study period 295 patients fulfilled the inclusion criteria. Of these, 270 were recruited and 25 patients did not participate either because they declined consent (n = 21), or because they had left the hospital by the time the culture became positive and could not be contacted (n = 4). The 270 study patients had 335 sterile site cultures positive for *S. aureus*, of which 201 were pus specimens obtained by aspiration or from operative procedures, 125 were blood cultures, 5 were pleural fluid, 3 were cerebrospinal fluid (CSF), and one was peritoneal dialysate. Considering patients as the denominator, 100 patients (37%) had positive blood cultures of whom 14 had at least one other positive sterile site culture from an identified focus of infection, and 170 patients had positive pus culture(s) alone.

All-cause mortality at 12 weeks was 70/270 (26%), of which 55/270 (20%) were attributable to *S. aureus* infection. Overall mortality for bacteraemic patients was 53%, compared with 10% in patients without a positive blood culture (p<0.001). Death occurred in 14/87 (16%) children and 56/183 (31%) adults (p = 0.01), of which 14% and 25% were attributable to *S. aureus*, respectively. The majority of infections (191, 71%) were community-acquired, of whom 34 (18%) died (Table 1). The remainder represented 27 (10%) cases of non-nosocomial healthcare-associated infection of whom 9 (33%) died, and 52 (19%) cases of nosocomial infection of whom 27 (52%) died (p<0.001 for comparison of 3 groups). Most of the 200 patients who survived to follow up at 12 weeks were cured (n = 189). The remaining 11 patients had a range of unresolved *S. aureus* infections: empyema (n = 2), diabetic foot infection (n = 2), infected orthopaedic material (n = 2), infected pacemaker with endocarditis (n = 1), osteomyelitis plus pyomyositis with (n = 1) or without (n = 1) septic arthritis, septic arthritis alone (n = 1) or orbital abscess (n = 1).

**Table 1:** Association between patient characteristics and outcome for 270 patients with *S. aureus* infection.

	Overall (n = 270)	Survivors (n = 200)	All-cause deaths (n = 70)	p value* ^1^
	*Number, % of overall patients*	*Number, % of survivors*	*Number, % of all deaths*	
**Demographics**				
Age (years); median (IQR)	39 (14–60)	35 (13–53)	55 (32–70)	**<0.001**
Sex (male)	163 (60%)	121 (61%)	42 (60%)	>0.999
Manual labour* ^2^ (if aged over 16 years)	147 (77%) (n = 191)	101 (75%) (n = 134)	46 (81%) (n = 57)	0.459
- Rice farming* ^2^	102 (53%) (n = 191)	72 (54%) (n = 134)	30 (53%) (n = 57)	>0.999
**Co-morbidities**				
Prematurity/Very low birth weight	7 (3%)	2 (1%)	5 (7%)	**0.014**
Underlying medical conditions* ^3^	131 (49%)	81 (41%)	50 (71%)	**<0.001**
- Diabetes mellitus	42 (16%)	31 (16%)	11 (16%)	>0.999
- Immunosuppression* ^4^	30 (11%)	20 (10%)	10 (14%)	0.377
- Renal disease* ^5^	25 (9%)	12 (6%)	13 (19%)	**0.003**
- Cardiac disease* ^6^	24 (9%)	5 (3%)	19 (27%)	**<0.001**
- Lung disease* ^7^	16 (6%)	8 (4%)	8 (11%)	**0.036**
**Source of infection**				
Community-acquired	191 (71%)	157 (79%)	34 (49%)	
Non-nosocomial healthcare-associated	27 (10%)	18 (9%)	9 (13%)	**<0.001**
Nosocomial	52 (19%)	25 (13%)	27 (39%)	
**Drug resistance**				
MRSA	42 (16%)	19 (10%)	23 (33%)	**<0.001**
**Interventions**				
Effective antibiotic therapy without delay	220 (81%)	175 (88%)	45 (64%)	**<0.001**
Procedure for infectious source control	184 (68%)	162 (81%)	22 (31%)	**<0.001**

Data are number (%) unless otherwise stated.

* ^1^p value for the comparison between all-cause deaths and survivors.

* ^2^Denominator for occupation is number of patients over the age of 16 years which is given in each square.

* ^3^Past medical history of any underlying chronic medical conditions reported by the patient/relative or recorded in the medical notes.

* ^4^Immunosuppression from HIV (5 untreated, 3 on anti-retroviral therapy), chemotherapy (n = 3), untreated leukaemia (n = 1), radiotherapy (n = 1) or immunosuppressive medication including prednisolone more than 30 mg/day for more than 1 week (n = 17).

* ^5^Renal disease included end stage renal failure on long-term dialysis (n = 3; 2 on haemodialysis, 1 on peritoneal dialysis) and chronic renal failure (not on dialysis) due to diabetes mellitus (n = 14), systemic lupus erythematosus (n = 1), multiple myeloma (n = 1), glomerulonephritis (n = 1) or an unknown aetiology (n = 5).

* ^6^Cardiac disease comprised congenital heart disease (n = 4), valvular heart disease including rheumatic heart disease (n = 8), ischaemic heart disease (n = 8), or arrhythmias including heart block requiring pacemaker (n = 4).

* ^7^Lung disease comprised previously treated tuberculosis (n = 9), previous empyema (n = 1), lung cancer (n = 2), long-term tracheostomy (n = 1), chronic obstructive pulmonary disease (n = 2) or asthma (n = 1).

Patient characteristics and their association with outcome are summarised in Table 1. Age on the day the first culture positive for *S. aureus* was taken ranged from 1 day to 92 years (median 39 years). Overall and attributable mortality were significantly associated with increasing age (p<0.001 for both), and overall mortality but not attributable mortality was associated with prematurity (p = 0.014). The presence of pre-existing cardiac, renal or lung disease was significantly associated with all-cause mortality and pre-existing cardiac and renal disease with attributable mortality.

Most of the 270 patients (n = 231, 86%) had one or more identifiable extra-vascular sites of infection, while no site of infection was identified other than positive blood culture in the remaining 39 cases (Table 2). Superficial and deep abscesses accounted for the majority of infections (34% and 17%, respectively), and were strongly associated with a survival benefit (p<0.001 and p = 0.004, respectively). Conversely, respiratory infections and having a blood culture positive for *S. aureus* were strongly associated with all-cause and attributable death (p<0.001 for all comparisons). Patients with an identified extra-vascular site of infection were less likely to die than those patients with bacteraemia alone (all-cause 20% vs. 59%, p<0.001; attributable 16% vs. 56%, p<0.001).

**Table 2:** The range of sites of infection in patients and outcome associated with each clinical presentation.

Clinical presentations	Total no. of sites (n = 264)	Total no. of patients (n = 270)	Survivors (n = 200)	All-cause deaths (n = 70)	p value* ^1^
	*Number, % of all sites*	*Number, % of overall patients*	*Number, % of survivors*	*Number, % of all deaths*	
**Pattern of disease**					
Bacteraemia only, no identified site of infection		39 (14%)	16 (8%)	23 (33%)	
1 identified site of infection		200 (74%)	157 (79%)	43 (61%)	**<0.001**
>1 identified site of infection		31 (11%)	27 (14%)	4 (6%)	
Blood culture positive		100 (37%)	47 (24%)	53 (76%)	**<0.001**
**Superficial abscesses** (skin and soft tissue)	89 (34%)	83 (31%)	82 (41%)	1 (1%)	**<0.001**
**Deep abscesses** * ^2^	46 (17%)	44 (16%)	40 (20%)	4 (6%)	**0.004**
**Other skin and soft tissue infections** * ^3^	31 (12%)	30 (11%)	19 (10%)	11 (16%)	0.184
**Bone and joint infections**	30 (11%)	27 (10%)	24 (12%)	3 (4%)	0.068
- Septic arthritis	16				
- Osteomyelitis	8				
- Diabetic foot infection	6				
**Prosthetic material infections**	24 (9%)	24 (9%)	17 (9%)	7 (10%)	0.807
- Orthopaedic material* ^4^	9				
- Intravenous device* ^5^	8				
- Pacemaker	3				
- Meningitis related to ventriculostomy drain	2				
- Arteriovenous graft	1				
- Peritonitis from peritoneal dialysis	1				
**Respiratory infections**	23 (9%)	22 (8%)	6 (3%)	16 (23%)	**<0.001**
- Pneumonia	12				
- Empyema	11				
**Endocarditis** * ^6^	8 (3%)	8 (3%)	6 (3%)	2 (3%)	>0.999
**Other infections** * ^7^	7 (3%)	7 (3%)	4 (2%)	3 (4%)	0.380
**Post-operative infections** * ^8^	6 (2%)	6 (2%)	3 (2%)	3 (4%)	0.182

* ^1^p value for the comparison between all-cause deaths and survivors.

* ^2^Site of deep abscesses were muscle (n = 20), retroperitoneal space (n = 7), parotid gland (n = 7), liver (n = 3), lung (n = 2), epidural space (n = 2), eye (n = 2), oropharynx (n = 2) and spleen (n = 1).

* ^3^Other skin and soft tissue infections includes: necrotising fasciitis (n = 9), bedsore(s) (n = 6), pustules and carbuncles (n = 5), infected wound from trauma (n = 3), infected wound from tophi (n = 2), gangrene (n = 2), cellulitis (without other skin or soft tissue lesion) (n = 2) and infection of exfoliated skin following a severe drug reaction (n = 2).

* ^4^Orthopaedic material includes: internal fixation metalwork (n = 8) and a hip replacement (n = 1).

* ^5^Intravenous devices were peripheral cannulas (n = 4), central catheters (n = 3) and an umbilical catheter (n = 1).

* ^6^Endocarditis from transthoracic echocardiographic evidence of vegetations (n = 7); 1 case clinically but died prior to echocardiogram.

* ^7^Other infections include: urinary tract infection (n = 3), tenosynovitis (n = 2), Lemierre's syndrome (n = 1) and corneal ulcer (n = 1).

* ^8^Post-operative infections include: mediastinitis (n = 4; 3 following mitral valve replacement and 1 after coronary artery bypass graft), meningitis from infected bone flap surgical wound (n = 1) and abdominal wound (n = 1).

**Figure 1:**
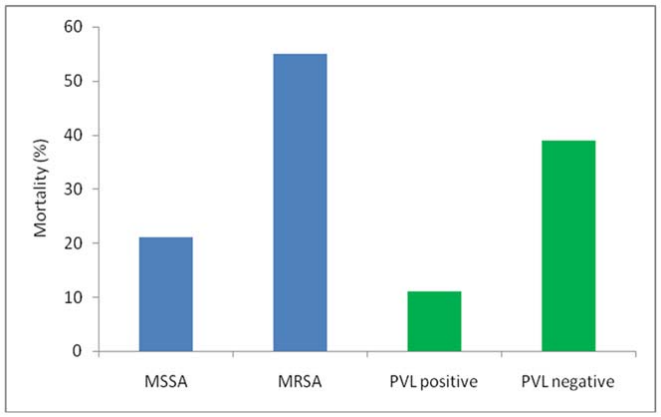
Higher all-cause mortality associated with methicillin-resistant *S. aureus* (MRSA) but not with Panton-Valentine Leukocidin (PVL). Patients infected by MRSA had a greater all-cause mortality compared with patients infected by methicillin-susceptible *S. aureus* (MSSA) (p<0.001). Conversely, patients infected by PVL gene-positive *S. aureus* had a lower all-cause mortality compared with patients infected by PVL gene-negative *S. aureus* (p<0.001), an association that remained after adjustment for MRSA (p = 0.001).

MRSA accounted for 16% (n = 42) of infections overall, rising to 26% in those patients with bacteraemia. There was no significant difference in the MRSA rates between adults and children. No community-acquired MRSA was identified, the MRSA infections being defined as nosocomial (n = 29, 69%), or non-nosocomial healthcare-associated (n = 13, 31%). MSSA infections were predominantly community-acquired (84%, n = 191), the remainder being nosocomial (n = 23, 10%) or non-nosocomial healthcare-associated (n = 14, 6%). MRSA infection was positively associated with all-cause death (p<0.001) (Figure 1), and the association remained highly significant for attributable mortality (p = 0.001).

**Figure 2:**
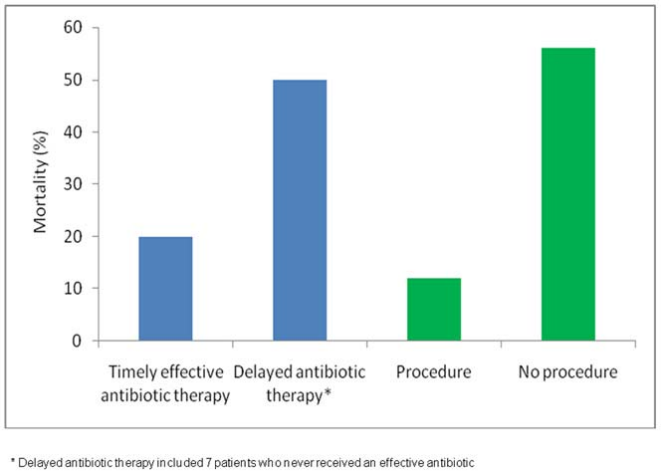
Timely effective antibiotic therapy and procedures for infectious source control significantly improved outcome. Administration of an effective antibiotic on the same day as the positive culture was taken significantly reduced all-cause mortality (p<0.001), as did undergoing a procedure for infectious source control (p<0.001).

Antibiotic therapy was prescribed to 269/270 patients, the single exception being a patient who was discharged from hospital prior to the culture results becoming available. The majority (n = 253, 94%) of patients commenced antibiotics on or before the day their positive culture was taken. Of these, 220 patients (87%) received an empiric antibiotic that covered their infecting strain of *S. aureus* as judged by *in vitro* susceptibility testing. Of the remainder, 42 patients had a delay in starting antibiotic therapy to which the organism was susceptible (median delay 3 days, IQR 2–4 days), and 7 patients never received an effective antibiotic. A delay in receiving effective antibiotic therapy or never receiving effective therapy was significantly associated with an increased all-cause mortality (p<0.001) (Figure 2) and attributable mortality (p<0.001).

A procedure for infectious source control was performed in 184 patients (68%). This was associated with a significantly improved outcome (p<0.001) (Figure 2). The procedures were: needle aspiration (n = 10), incision and drainage (n = 109), debridement (n = 35), joint washout (n = 8), removal of prosthesis (n = 7), thoracotomy/decortication (n = 4), nephrectomy (n = 3), laminectomy (n = 2), chest drain (n = 2), above knee amputation (n = 1), fasciotomy (n = 1), ethmoidectomy (n = 1) and craniectomy with removal of infected bone flap (n = 1).

*S. aureus* isolates from 248 patients were available for PCR, of which 122 (49%) were PVL gene-positive. All of the PVL gene-positive isolates were MSSA. The white cell count at the time of culture was not significantly different between cases infected with PVL gene-positive isolates and cases infected with PVL gene-negative isolates (median 15.30×10^9^/L vs. 14.69×10^9^/L, respectively, p = 0.38). The distribution of clinical presentations in relation to PVL is shown in Table 3. PVL gene-positive isolates were significantly more likely to cause skin and soft tissue abscesses (p<0.001) and deep abscesses (p = 0.001) than isolates that were PVL gene-negative. A significant negative association was observed between the presence of PVL genes and bacteraemia without a localising site of infection (p<0.001), prosthetic material infections (p<0.001) and other skin and soft tissue infections (p = 0.026). Additionally, there was a highly significant negative association between the presence of PVL genes and all-cause mortality (p<0.001) (Figure 1), as well as *S. aureus*-attributable mortality (p<0.001). This association remained highly significant when adjusted for MRSA cases (p = 0.001). The outcome from pneumonia was poor regardless of PVL, with a mortality rate of 80% for PVL gene-positive cases and 83% for PVL gene-negative cases.

**Table 3:** Association between clinical presentation and presence of Panton-Valentine Leukocidin (PVL) in the infecting isolate for 248 patients with *S. aureus* infection.

Clinical presentations	Total no. of patients (n = 248)	PVL positive (n = 122)	PVL negative (n = 126)	p value* ^1^
Bacteraemia only, no identified site of infection	29 (12%)	3 (2%)	26 (21%)	**<0.001**
Superficial abscesses	80 (32%)	60 (49%)	20 (16%)	**<0.001**
Deep abscesses	43 (17%)	31 (25%)	12 (10%)	**0.001**
Other skin and soft tissue infections	28 (11%)	8 (7%)	20 (16%)	**0.026**
Bone and joint infections	25 (10%)	12 (10%)	13 (10%)	>0.999
Prosthetic material infections	24 (10%)	2 (2%)	22 (17%)	**<0.001**
Respiratory infections	19 (8%)	8 (7%)	11 (9%)	0.635
- Pneumonia* ^2^	11 (4%)	5 (4%)	6 (5%)	>0.999
Endocarditis	7 (3%)	2 (2%)	5 (4%)	0.447
Other infections	7 (3%)	5 (4%)	2 (2%)	0.275
Post-operative infections	4 (2%)	0	4 (3%)	0.122

Data are number (%). Denominator is the site of clinical presentation rather than patients.

* ^1^p value for the comparison between PVL positive and PVL negative cases.

* ^2^Subset of respiratory infections with pneumonia.

All variables in Tables 1 and 2 found to be significantly associated with all-cause and attributable mortality on univariable testing and with 5 or more events (deaths) together with the presence of PVL genes, were further analysed using multiple logistic regression to give the final models shown in Table 4. Cardiac, renal and lung disease were considered as separate variables for all-cause mortality, and cardiac and renal disease for attributable mortality. Only cardiac disease remained in each of the final models. Age, underlying cardiac disease and respiratory infection were positively associated with all-cause mortality, while one or more abscesses (deep and superficial combined) as the presenting clinical feature and a procedure for infectious source control were negatively associated with all-cause mortality. An analysis of *S. aureus*-attributable mortality demonstrated the same associations. Underlying cardiac disease and respiratory infection had the highest odd ratios for both all-cause mortality and *S. aureus*-attributable mortality.

**Table 4:** Significant risk factors for mortality from *S. aureus* infection from multiple logistic regression analysis.

All-cause mortality	Odds ratio (95% CI)* ^1^	p value* ^2^
Age	1.03 (1.01–1.05)	<0.001
Underlying cardiac disease	10.43 (2.96–36.70)	<0.001
Respiratory infection	6.46 (2.13–19.62)	0.001
Abscesses (superficial and deep combined)	0.13 (0.04–0.40)	<0.001
Procedure for infectious source control	0.22 (0.10–0.51)	<0.001

* ^1^95% confidence intervals.

* ^2^p value from Likelihood ratio test.

## Discussion

Practical, inexpensive solutions are needed to improve the outcome of bacterial sepsis in developing country settings. Time to the first dose of effective antibiotics has been related to outcome for a range of bacterial infectious diseases in the developed world [13], [27], and held true in this study of *S. aureus* infection. Our hospital-based study conducted in provincial Thailand is relevant to the numerous developing countries with growing economies and expanding healthcare facilities, many of which are in South and East Asia, where it is possible to identify patients with suspected bacterial sepsis, administer empiric, broad-spectrum antibiotics, and drain pus collections. Identification of abscesses and drainage of pus is fundamental to sepsis control, and the use of available imaging to investigate patients with *S. aureus* sepsis and guide drainage procedures should be a priority. The higher mortality observed in our study for patients with bacteraemia in whom a site other than bloodstream was not identified is consistent with the higher mortality in patients who had a focus but no procedure for source control, and with the survival benefit for patients who underwent a procedure. The risk factors for death identified in this study are comparable to those reported elsewhere. An awareness of those patient groups at high risk for death can have considerable clinical utility in settings where healthcare facilities are available but limited and healthcare workers have a large patient caseload.

Nosocomial infection and drug resistance are the focus of enormous research efforts and expenditure in the developed world. In developing countries worldwide, these are generally poorly understood although there is wide variability. Nosocomial infection is probably common, the reasons for which may include lack of hand wash basins and/or hand washing, overcrowding in hospital wards and clinics, lack of infection control training or policies, an inability to isolate specific patients, and a lack of awareness of the presence of nosocomial infection resulting from a lack of diagnostic microbiology facilities. Drug resistance is also likely to be underestimated in regions with little or no microbiology capacity, and is probably driven in such settings by the availability of a range of over-the-counter antibiotics. The importance of both nosocomial infection and drug resistance were highlighted by our study. Nearly one third of infections were nosocomial or non-nosocomial healthcare-associated, and patients in these groups had significantly higher mortality rates for both all-cause and *S. aureus*-attributable deaths. Infection with MRSA was either nosocomial or non-nosocomial healthcare-associated, and was independently associated with all-cause and attributable mortality. Those patients affected by healthcare-associated infection often have other risk factors for death such as prematurity, old age, underlying medical conditions or have undergone recent surgery and the high mortality rate is not surprising. However, these infections and infection-related deaths are potentially preventable, and there is a clear need for studies in such settings that evaluate low cost preventive interventions such as a hand hygiene campaign associated with provision of improved hand washing facilities or alcohol hand gel.

Nearly half of isolates causing *S. aureus* infection in patients presenting to our hospital were PVL gene-positive, which is significantly higher than that for previous reports for infecting isolates amongst blood culture and skin infection samples from Malaysia (5%) [28], skin infections in Bangladesh (14.3%) [29], and blood culture (2.1%) and soft tissue infection samples (38.9%) from the Netherlands [30]. This may imply that isolates that are PVL gene-positive are more likely to cause infection than isolates that are PVL gene*-*negative (that is, the rate of PVL gene-positivity is lower in carriage than invasive strains). This could either be a function of PVL per se, or could relate to one or more alternative genes associated with disease acquisition that is in genetic linkage with the PVL genes. Such questions will be addressed by us during community carriage studies to determine rates of PVL gene positivity, together with genotyping of carriage and invasive isolates.

The role of PVL as a putative virulence determinant is a hotly debated topic within the staphylococcal community, and this led us to examine the association between this and both clinical presentations and outcome. Our study design was robust in that we recruited all patients with *S. aureus* infection who required hospital treatment. Previous reports have been based on patient subsets determined by clinical presentation [19], culture sample type [17] or by being sent to a reference laboratory [18]. The single most important finding was that PVL gene-positive isolates were strongly associated with patient survival compared to PVL gene-negative isolates. This may be explained in large part by the fact that PVL gene-positive isolates were associated with skin and soft tissue abscesses, an association reported previously [16], [18], [31] and a clinical manifestation associated with low morbidity. Community-acquired PVL-associated necrotising pneumonia affecting previously fit people is often fatal and has gained considerable notoriety. In our study we identified 3/270 patients (1%) with PVL gene-positive, community-acquired pneumonia in previously healthy individuals. None of these patients had haemoptysis, which is deemed a major diagnostic criterion for PVL-associated necrotising pneumonia [32], although none of the prospective cases in the original paper by Gillet et al describing PVL-associated necrotising pneumonia were described as having had haemoptysis [19]. Two out of 3 of these patients died, but this was in the context of a very high mortality of >80% for *S. aureus* pneumonia overall. Although further cohort studies of unselected patients are needed to determine the role of PVL as a virulence factor in other geographic settings, the current study provides no support for rapid PVL testing in this and other developing country settings, where the focus should firmly rest with clinical interventions that save lives.

In summary, this prospective cohort study has identified risk factors for mortality in *S. aureus* disease and highlights clinical strategies that reduce death. *S. aureus* is gaining increasing recognition as an important pathogen in developing country settings. Initiatives are required to optimise the use of available resources and affordable interventions for patients with *S. aureus* infection in the developing world.
